# A cross-sectional study of relationships between social risks and prevalence and severity of pediatric chronic conditions

**DOI:** 10.1186/s12887-023-03894-6

**Published:** 2023-03-08

**Authors:** Annelise Brochier, Emily Messmer, Mikayla Gordon Wexler, Stephen Rogers, Erika Cottrell, Yorghos Tripodis, Arvin Garg

**Affiliations:** 1 Department of Pediatrics, Boston Medical Center, 801 Albany St. Floor 2N, Boston, MA 02119 USA; 2 Quality and Patient Experience, Mass General Brigham, 399 Revolution Dr., Somerville, MA 02145 USA; 3 Icahn School of Medicine at Mount Sinai, 1 Gustave L. Levy Pl., New York, NY 10029 USA; 4 Department of Pediatrics, Children’s Hospital of Philadelphia, 3401 Civic Center Blvd., Philadelphia, PA 9104 USA; 5 OCHIN, Inc., PO Box 5426, Portland, OR 97228 USA; 6 Oregon Clinical & Translational Research Institute, Oregon Health & Science University, 3250 Southwest Sam Jackson Park Rd., Portland, OR 97329 USA; 7 Department of Biostatistics, Boston University School of Public Health, 801 Massachusetts Ave., 3rd Floor, Boston, MA 02118 USA; 8 Child Health Equity Center, Department of Pediatrics, UMass Chan Medical School, UMass Memorial Children’s Medical Center, 55 N Lake Ave., Worcester, MA 01655 USA

**Keywords:** Social determinants of health, Social risks, Attention deficit hyperactivity disorder, Autism spectrum disorder, Asthma, Obesity

## Abstract

**Background:**

To examine the differential relationships between seven social risk factors (individually and cumulatively) with the prevalence and severity of asthma, attention deficit hyperactivity disorder (ADHD), autism spectrum disorder (ASD), and overweight/obesity in children.

**Methods:**

Using the 2017–2018 National Survey of Children's Health, we examined associations between social risk factors (caregiver education, caregiver underemployment, discrimination, food insecurity, insurance coverage, neighborhood support, and neighborhood safety) and the prevalence and severity of asthma, ADHD, ASD, and overweight/obesity. We used multivariable logistic regression to assess the relationship between individual and cumulative risk factors with each pediatric chronic condition, controlling for child sex and age.

**Results:**

Although each social risk factor was significantly associated with increased prevalence and/or severity of at least one of the pediatric chronic conditions we investigated, food insecurity was significantly associated with higher disease prevalence and severity for all four conditions. Caregiver underemployment, low social support, and discrimination were significantly associated with higher disease prevalence across all conditions. For each additional social risk factor a child was exposed to, their odds of having each condition increased: overweight/obesity (aOR: 1.2, 95% CI: [1.2, 1.3]), asthma (aOR: 1.3, 95% CI: [1.2, 1.3], ADHD (aOR: 1.2, 95% CI: [1.2, 1.3]), and ASD (aOR: 1.4, 95% CI: [1.3, 1.5]).

**Conclusions:**

This study elucidates differential relationships between several social risk factors and the prevalence and severity of common pediatric chronic conditions. While more research is needed, our results suggest that social risks, particularly food insecurity, are potential factors in the development of pediatric chronic conditions.

## Background

In the United States (US), more than 40% of children have a chronic condition such as asthma, attention deficit hyperactivity disorder (ADHD), autism spectrum disorder (ASD) or obesity [1]. Social determinants of health (SDOH), the social circumstances in which people are born, live, work, and age, are key drivers of health and disease—especially for children, who are particularly vulnerable to adverse SDOH, also termed social risk factors, such as poor neighborhood conditions, housing instability, and food insecurity (FI) [2–5]. Yet, it remains unclear how multiple social risks differentially impact the prevalence and severity of common childhood chronic diseases. It is hypothesized, and demonstrated in animal studies, that social risks can act as toxic stressors that disrupt the hypothalamic-pituitary-adrenocortical axis and cause epigenetic changes, leading to the development of chronic diseases [6–8]. The cumulative risk model posits that having multiple risk factors increases the likelihood of developing pediatric disease and developmental and behavioral problems [9]. Additionally, longitudinal studies demonstrate that increased time spent living in poverty in childhood is associated with elevated cortisol and dysregulated cardiovascular response, mediated by cumulative risk exposure; however, little is known about the relationship between specific adverse SDOH and common pediatric conditions [9, 10].

Previous studies have demonstrated that FI is associated with worse general health as well as acute and chronic health conditions in children and adults [11]. Increased asthma morbidity in children is associated with poverty, poor housing, less neighborhood safety, and low social and community support [12]. Furthermore, an analysis of the 2003–2004 National Survey of Children’s Health (NSCH) dataset found that Black, Latinx, and low-income families reported lower prevalence of ASD than White and middle-to-high-income families, yet reported higher ASD severity [13]. A systematic review of socioeconomic variables and chronic conditions found that parental socioeconomic status was a determinant of health-related quality of life for children with a chronic condition [14]. Understanding the differential association of various social risk factors with common pediatric chronic conditions may inform management strategies for chronic diseases (e.g., screening for social risk factors at healthcare visits for children with asthma), and inform social and health policies.

To advance our understanding of SDOH and their influence on the development of childhood chronic conditions, we used nationally representative data to assess the associations between multiple social risks—individually and cumulatively—with prevalence and severity of asthma, ADHD, ASD, and overweight/obesity.

## Methods

### Data set

We conducted a secondary data analysis of the 2017–2018 NSCH—a nationally representative survey conducted annually by the Maternal and Child Health Bureau and the US Census Bureau [15]. The NSCH is administered via web-based and mail instruments. US households are randomly contacted by mail to identify those with one or more children under 18 years old. In each eligible household, one child is randomly selected as the subject of the survey and the respondent is any adult in the household who has knowledge of the child’s health and health care; of note, we refer to these respondents as caregivers hereafter. The NSCH collects data on children’s physical and mental health; access and quality of health care; social risks including family, neighborhood, and school characteristics; as well as adverse childhood experiences. The 2017–2018 two-year combined NSCH dataset comprises 52,129 completed surveys, and includes 4,041 children with asthma, 4,540 children with ADHD, 1,321 children with ASD, and 7,255 children with overweight/obesity.

### Independent variables

**Fig. 1:**
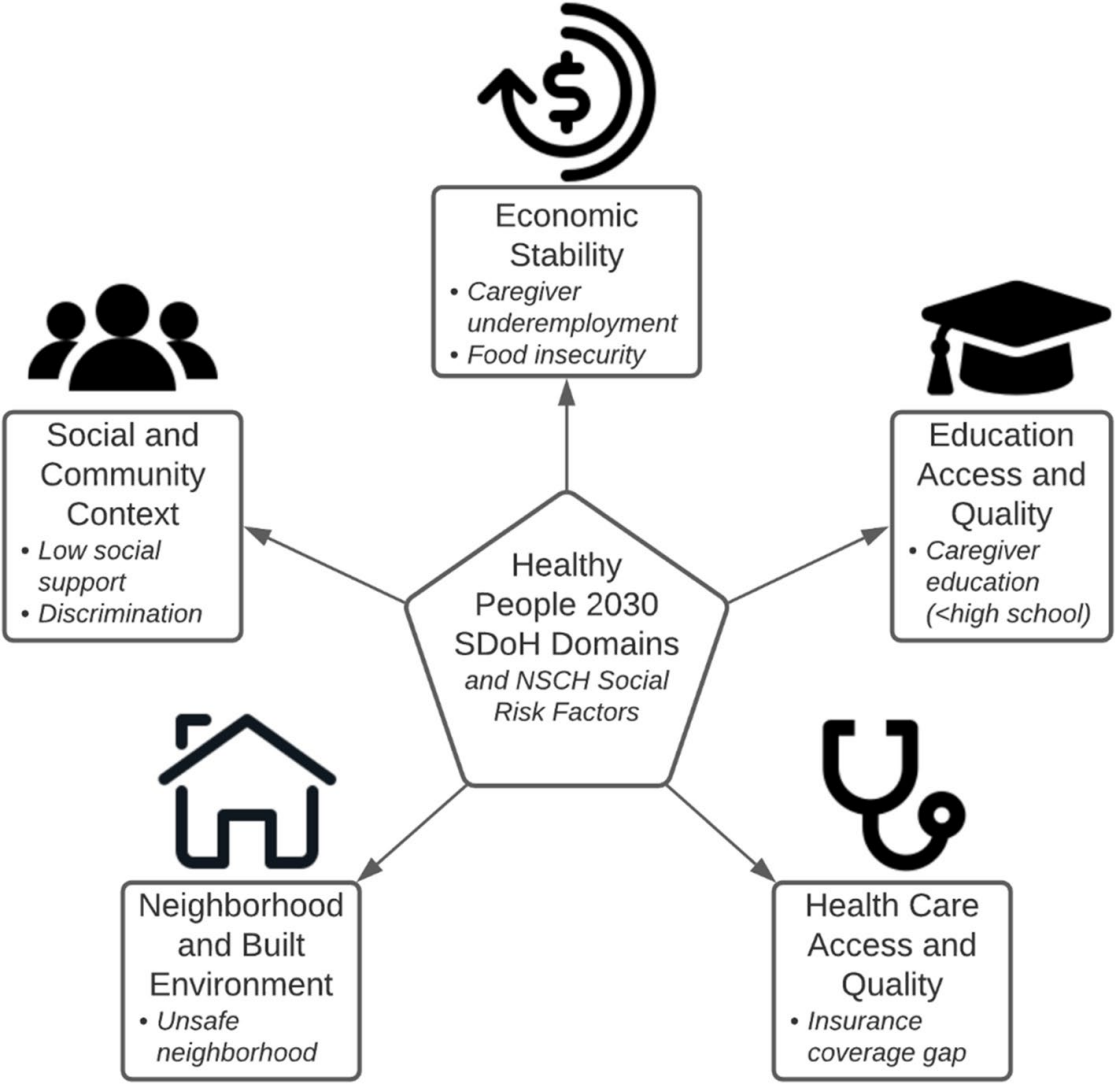
Social risks from the National Survey of Children’s Health (NSCH) mapped to Healthy People 2030’s social determinants of health (SDoH) domains.

The US Department of Health and Human Services’ Healthy People 2030 initiative classifies SDOH by five different domains: economic stability; education access and quality; health care access and quality; neighborhood and built environment; and social and community context [16]. We selected seven social risk factors (caregiver education less than high school, caregiver underemployment, child’s experience of discrimination, household FI, gap in child’s insurance coverage, neighborhood social support, and safety of child’s neighborhood) assessed through the below NSCH questions that map to relevant Healthy People 2030 SDOH domains (Fig. 1). Consistent with prior studies, we used NSCH-established scoring criteria to define each social risk factor and categorize each child’s exposure [17–19].

#### Economic stability

The two social risk factors we mapped to economic stability were “caregiver underemployment” and “food insecurity”. Children who lived in households where none of the adult primary caregivers were employed at least 50 of the last 52 weeks were categorized as having “caregiver underemployment.”

Respondents were also asked to describe their “ability to afford the food the child’s family needed in the past 12 months”. If the caregiver responded with “We could always afford enough to eat but not always the kinds of food we should eat”. “Sometimes we could not afford enough to eat,” or “Often we could not afford enough to eat,” the child was categorized “food insecure”.

#### Education access and quality

To measure education access and quality, we examined caregivers’ highest level of educational attainment. We categorized children whose caregivers’ education level was “Less than high school” as having the “caregiver education” social risk factor.

#### Health and healthcare

Children whose caregivers indicated that the child did not have consistent health insurance coverage during the preceding 12 months were categorized as having the “insurance coverage gap” risk factor, which we mapped to the health and healthcare SDOH domain.

#### Neighborhood and built environment

When caregivers indicated that they “somewhat disagree” or “definitely disagree” with the statement “the child is safe in our neighborhood,” we considered the child to have the “unsafe neighborhood” social risk factor within the neighborhood and built environment SDOH domain.

#### Social and community context

We mapped two social risk factors to the social and community context domain: low social support and discrimination. The NSCH measures neighborhood support based on responses to three statements: “People in the neighborhood help each other out”; “We watch out for each other’s children in this neighborhood”; and “When we encounter difficulties, we know where to go for help in our community.” Only surveys with valid responses to all three questions were included in the denominator for the “neighborhood support” variable. If the caregiver did not respond “definitely agree” to at least one of the items and “somewhat agree” or “definitely agree” to the other two items, we categorized the child as having low social support.

The NSCH also inquires about 9 adverse childhood experiences, one of which is “the child was treated or judged unfairly because of his/her race or ethnic group.” If the caregiver responded “Yes” to this specific adverse childhood experience, we considered the child to have experienced discrimination.

### Dependent variables

We assessed the prevalence and severity of the four most prevalent chronic childhood conditions available in the NSCH dataset: asthma, ADHD, ASD and overweight/obesity. Respondents were asked if the child currently has asthma, ADHD, or ASD, and if so, whether the severity of each condition was “mild” or “moderate/severe”.

Overweight/obesity was assessed using the child’s body mass index (BMI) percentile, which was calculated from the respondent’s report of child height, weight, and age. The child was considered overweight if their BMI was in the 85^th^ to 94^th^ percentile, and obese if their BMI was at or above the 95^th^ percentile. The NSCH only calculates BMI for children ages 10 through 17, therefore our denominator for the overweight/obesity prevalence and severity variables only included these children. In our analyses, the “severity” of overweight/obesity was considered “mild” if the child was overweight, and “moderate/severe” if the child was obese.

### Statistical analysis

We examined sociodemographic characteristics and social risk factors via descriptive statistics. We used multivariable logistic regression to assess the relationship between individual and cumulative social risk factors with the prevalence of each of the four chronic conditions, and repeated that analysis with each social risk factor and the severity of each condition. We adjusted for child’s sex and age in our analysis; we purposefully did not adjust for other sociodemographic characteristics such as household income and race/ethnicity due to their collinearity with social risk factors, which would have led to over-adjustment of our models.

We performed all statistical analyses using the SAS software version 9.4 (SAS Institute, Inc., Cary, NC) using survey-specific SAS procedures for weighting, clustering, and stratification in the survey design (PROC SURVEYMEANS, PROC SURVEYFREQ and PROC SURVEYLOGISTIC). Adjusted odds ratios (aORs), 95% confidence intervals (CIs), and *P* values were calculated for each model. Statistical significance was defined as *p* < 0.05.

## Results

### Sociodemographic characteristics

Overall, for the 2017–2018 combined NSCH dataset, a total of 52,129 surveys were completed with overall weighted response rates of 37.4% in 2017 and 43.1% 2018. The majority of respondents identified their children as male (51.1%), non-Hispanic White (50.7%), and between the ages of 12–17 years (34.0%). Over half (adjusted percentage: 63%) of the children had at least one of the seven risk factors studied. Overall, 38.2% of children lived in an environment with low social support, 26.1% were food insecure, 5.8% had inconsistent healthcare insurance coverage over the preceding 12 months, 3.1% experienced discrimination, and 3.1% lived in an unsafe neighborhood. Additionally, 7.3% of children had no caregivers who were employed fulltime, and 2.3% of children had no caregivers with at least a high school diploma or equivalent.

The most prevalent chronic condition in our sample was overweight/obesity (30.8%), followed by ADHD (8.7%), asthma (7.6%), and ASD (2.9%). Table 1 includes the number and weighted percentages of children’s sociodemographic characteristics by chronic condition.

**Table 1:** Sociodemographic characteristics and social risk factors, by chronic condition

	**Asthma** n, % (SE)	**Attention Deficit Hyperactivity Disorder** n, % (SE)	**Autism Spectrum Disorder** n, % (SE)	**Overweight/ Obesity** n, % (SE)
**Total**	4041, 7.6	4540, 8.7	1321, 2.9	7255, 30.8
**Sex**				
Female	1766, 44.5 (1.7)	1444, 32.4 (1.6)	280, 22.1 (2.9)	3150, 47.4 (1.3)
Male	2275, 55.5 (1.7)	30,096, 67.6 (1.6)	1041, 77.9 (2.9)	4105, 52.6 (1.3)
**Age**				
0–5	527, 17.1 (1.3)	118, 3.5 (0.6)	140, 10,3 (1.5)	N/A
6–11	1390, 39.0 (1.7)	1743, 45.0 (1.6)	465, 46.2 (3.6)	1725, 26.8 (1.2)
12–17	2124, 43.9 (1.7)	2679, 51.5 (1.6)	716, 43.5 (3.5)	5530, 73.2 (1.2)
**Race**				
Asian, non-Hispanic	114, 3.0 (0.7)	71, 0.9 (0.2)	55, 3.7 (0.9)	249, 2.9 (0.4)
Black, non-Hispanic	518, 24.4 (1.5)	334, 16.8 (1.4)	88, 14.4 (2.3)	673, 17.8 (1.1)
Hispanic	526, 23.3 (1.8)	451, 19.9 (1.6)	173, 34.0 (4.0)	1005, 31.2 (1.4)
Multi-racial/Other, non-Hispanic	382, 7.0 (0.7)	366, 6.2 (0.6)	117, 4.8 (0.7)	551, 5.8 (0.5)
White, non-Hispanic	2501, 23.3 (1.8)	3318, 56.2 (1.6)	888, 43.1 (3.0)	4777, 42.2 (1.2)
**Social Risk Factors**				
Caregiver Education (< high school)	114, 9.5 (1.5)	115, 9.2 (1.5)	34, 12.2 (3.7)	303, 15.8 (1.4)
Caregiver Underemployment	494, 17.7 (1.4)	556, 16.4 (1.3)	192, 18.1 (2.4)	722, 14.0 (1.0)
Discrimination	244, 8.5 (1.1)	238, 8.0 (1.0)	69, 8.1 (1.6)	386, 7.6 (0.7)
Food Insecurity	1438, 42.1 (1.7)	1603, 42.6 (1.6)	502, 49.1 (3.6)	2556, 41.0 (1.4)
Insurance Coverage Gap	264, 8.9 (1.0)	256, 7.7 (0.8)	74, 8.3 (2.1)	506, 11.0 (0.9)
Low Social Support	1691, 50.1 (1.7)	2001, 49.9 (1.6)	677, 56.8 (3.4)	3041, 46.2 (1.4)
Unsafe Neighborhood	186, 8.1 (1.3)	206, 6.5 (0.8)	80, 9.0 (2.9)	255, 5.7 (0.6)
**Total Number of Social Risk Factors**				
0 Risk factors	1478, 28.3 (1.4)	1625, 28.2 (1.2)	400, 23.6 (2.4)	2622, 27.7 (1.1)
1 Risk factor	1305, 29.6 (1.5)	1519, 32.1 (1.4)	441, 23.5 (2.1)	2447, 31.1 (1.2)
2 Risk factors	802, 23.2 (1.6)	899, 21.8 (1.3)	307, 28.7 (3.1)	1448, 24.1 (1.3)
3 Risk factors	331, 12.6 (1.4)	367, 12.3 (1.4)	127, 19.3 (4.1)	559, 11.4 (0.9)
4 Risk factors	102, 5.5 (1.0)	98, 3.9 (0.6)	39, 4.2 (1.1)	151, 4.7 (0.7)
5 Risk factors	17, 0.5 (0.2)	27, 1.5 (0.4)	6, 0.6 (0.4)	23, 0.8 (0.3)
> 6 Risk factors	6, 0.4 (0.2)	5, 0.3 (0.2)	1, 0.0 (0.0)	5, 0.1 (0.1)

**Fig. 2:**
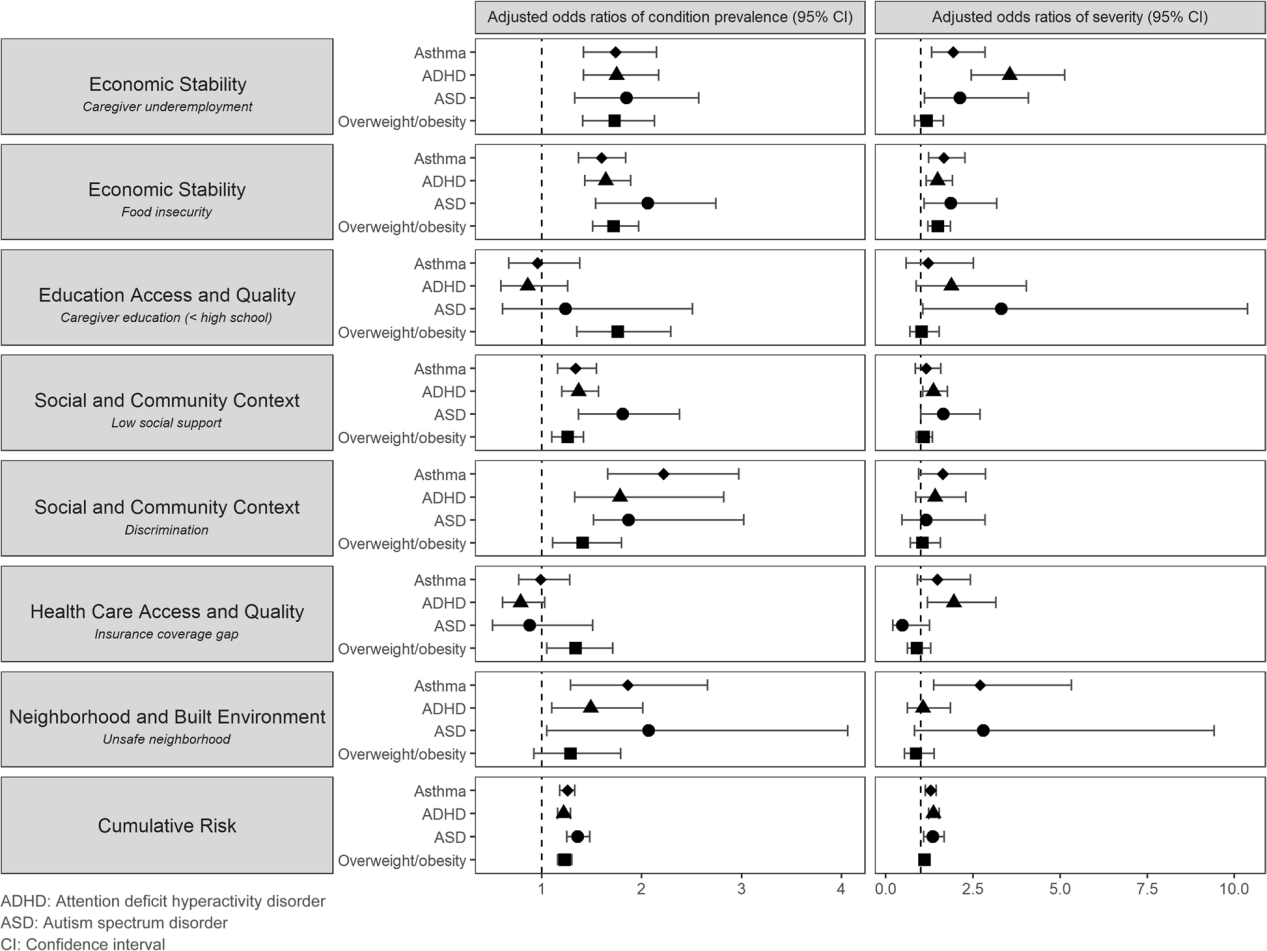
Adjusted odds ratios of condition prevalence and severity, by social risk factor, adjusted for child age and sex

Figure 2 plots the aORs and 95% CIs of condition prevalence and severity, grouped by social risk factor. Overall, within each individual social risk domain, differences in associations between the social risk and relative prevalence or severity of any of the chronic conditions were insignificant. For example, within “caregiver education,” the odds of severe asthma was not statistically significantly different from the odds of severe ADHD, ASD, or overweight/obesity.

### Economic Stability

Children whose caregivers were underemployed had significantly increased odds of being overweight/obese (aOR: 1.7; 95% CI: 1.4–2.1) and of having asthma (aOR: 1.7; 95% CI: 1.4–2.2), ADHD (aOR: 1.8; 95% CI: 1.4–2.2), or ASD (aOR: 1.9; 95% CI: 1.3–2.6) compared to children whose caregivers were employed. Caregivers’ underemployment was significantly associated with higher severity of every condition except overweight/obesity.

Compared to children who were not experiencing FI, children who were food insecure had increased odds of being overweight/obese (aOR: 1.7; 95% CI: 1.5–2.0) and of having asthma (aOR: 1.6; 5% CI: 1.4–1.8), ADHD (aOR: 1.6; 95% CI: 1.4–1.9), or ASD (aOR: 2.1; 95% CI: 1.5–2.7). Additionally, FI was associated with higher severity for all four chronic conditions.

### Education access and quality

Children whose caregivers’ highest level of educational attainment was less than high school had significantly increased odds of being overweight/obese (aOR: 1.8; 95% CI: 1.3, 2.3) and of moderate/severe ASD (aOR: 3.3; 95% CI: 1.1, 10.4).

### Health and healthcare

Lack of consistent health insurance was significantly associated with higher odds of overweight/obesity prevalence (aOR 1.3; 95% CI: 1.1, 1.7) and ADHD severity (aOR: 2.0; 95% CI: 1.2, 3.2).

### Neighborhood and built environment

Living in an unsafe neighborhood was significantly associated with higher odds of asthma (aOR: 1.9; 95% CI: 1.3, 2.7), ADHD (aOR: 1.5; 95% CI: 1.1, 2.0), and ASD (aOR: 2.1; 95% CI: 1.1, 4.1), as well as increased odds of moderate/severe asthma (aOR: 2.7; 95% CI: 1.4, 5.3).

### Social and community context

Low social support was significantly associated with all four chronic conditions; children with low social support had higher odds of being overweight/obese (aOR: 1.3; 95% CI: 1.1–1.4) and of having asthma (aOR: 1.3; 95% CI: 1.2–1.6), ADHD (aOR: 1.4; 95% CI: 1.2–1.6), or ASD (aOR: 1.8; 95% CI: 1.4–2.4). Low social support was significantly associated with increased odds of moderate/severe ADHD (aOR: 1.4; 95% CI: 1.1, 1.8) or ASD (aOR: 1.7; 95% CI: 1.0, 2.7).

Though discrimination was not significantly associated with increased severity of any of the four conditions, children who experienced discrimination had significantly higher odds of being overweight/obese (aOR: 1.4; 95% CI: 1.1, 1.8), and of having asthma (aOR: 2.2; 95% CI: 1.7, 3.0), ADHD (aOR: 1.8; 95% CI: 1.3, 2.8), or ASD (aOR: 1.9; 95% CI: 1.5–3.0).

### Cumulative impact

On average, for each additional social risk factor a child was exposed to, their odds of having a chronic condition increased; specifically, each additional risk factor was associated with increased odds of overweight/obesity (aOR: 1.2, 95% CI: 1.2, 1.3), asthma (aOR: 1.3, 95% CI: 1.2, 1.3), ADHD (aOR:1.2, 95% CI: 1.2, 1.3), and ASD (aOR: 1.4, 95% CI: 1.3, 1.5). Looking more granularly, the odds of overweight/obesity, asthma, and ADHD significantly increased from 0 to 1 risk factor and odds of overweight/obesity, asthma, and ASD significantly increased from 1 to 2 risk factors (Table 2). Among children with ADHD, their odds of moderate/severe ADHD significantly increased from 0 to 1 risk factors (aOR: 1.3, 95% CI: 1.0, 1.8) and from 2 to 3 risk factors (aOR: 2.0, 95% CI: 1.2, 3.5). Among children with ASD, those with 3 social risk factors had 5.7 times the odds of moderate/severe ASD compared to those with 2 social risk factors (95% CI: 2.3, 15.5).

**Table 2:** Adjusted odds ratios of chronic condition prevalence and severity by N social risk factors compared to N-1 social risk factors

**Number of social risks**	**Health outcomes**	**Adjusted odds ratio of condition prevalence** [95% confidence interval]	**Adjusted odds ratio of moderate/severe** [95% confidence interval]
1	Asthma	1.23 [1.05, 1.44]*	1.33 [0.94, 1.89]
	ADHD^†^	1.37 [1.18, 1.58]*	1.33 [1.01, 1.75]*
	ASD^‡^	1.17 [0.91, 1.52]	0.82 [0.49, 1.36]
	Overweight/Obesity	1.46 [1.27, 1.68]*	1.19 [0.93, 1.51]
2	Asthma	1.28 [1.05, 1.56]*	1.11 [0.72, 1.72]
	ADHD	1.08 [0.89, 1.29]	1.29 [0.91, 1.85]
	ASD	1.97 [1.46, 2.67]*	1.19 [0.64, 2.18]
	Overweight/Obesity	1.37 [1.44, 1.64]*	1.20 [0.90, 1.62]
3	Asthma	1.26 [0.92, 1.73]	1.56 [0.89, 2.72]
	ADHD	1.32 [0.96, 1.81]	2.03 [1.18, 3.49]*
	ASD	1.55 [0.88, 1.54]	5.72 [2.26, 15.49]*
	Overweight/Obesity	1.0 [0.8, 1.31]	1.03 [0.68, 1.56]
> 4	Asthma	1.46 [0.94, 2.25]	1.42 [0.61 3.28]
	ADHD	1.26 [0.84, 1.90]	0.73 [0.36, 1.47]
	ASD	0.64[0.81, 2.08]	0.53 [0.23, 1.22]
	Overweight/Obesity	1.43 [0.93, 2.21]	0.72 [0.40, 1.29]

^†^*ADHD* Attention deficit hyperactivity disorder.

^‡^*ASD* Autism spectrum disorder.

^*^p < 0.05.

## Discussion

In this nationally representative sample of US children, we found that several social risk factors (namely, caregiver underemployment, FI, low social support, and discrimination) were significantly associated with each pediatric chronic condition. Additionally, we found that, consistent with the cumulative risk model, children with multiple social risk factors (i.e., 3 vs. 1) had increased odds of each condition (and of worse condition severity) with each additional social risk. These results demonstrate a strong association between social risk factors and increased prevalence and severity of chronic conditions broadly, as well as differential associations across various SDOH domains and chronic conditions. For example, we found that social risk factors related to economic stability and social and community context were more strongly associated with condition prevalence than were social risks in the health care and education domains. Our findings align with and expand upon research demonstrating associations between individual and cumulative social risks with adult chronic conditions, including hypertension, diabetes, asthma, and depression [20, 21].

FI was the only social risk factor associated with increased prevalence and severity of all four chronic conditions. We classified households as having FI for three of four possible response options, encompassing a spectrum of food security from marginal (i.e., anxiety over food sufficiency) to very low (i.e., disrupted eating patterns and reduced food intake) [22]. The associations we found between FI with pediatric chronic conditions are consistent with a growing body of evidence linking even marginal FI with child health (e.g., asthma, anemia, obesity), health care utilization (e.g., forgone medical care), and adverse psychosocial and developmental outcomes [10, 23–25]. Researchers suggest these outcomes may be attributable to FI disrupting physiological stress-response systems by increasing children’s allostatic load; however, a systemic, pathophysiological framework to explain these results remains a gap in the literature [26, 27].

Caregiver unemployment—our second measure of economic instability—was also strongly associated with prevalence of all four chronic conditions, and severity of all except overweight/obesity. The strong associations between FI and caregiver unemployment with disease outcomes indicate that economic instability may have pernicious, short- and long-term harmful effects on child health [28, 29]. This is especially salient in the context of the COVID-19 pandemic, which has had severe economic ramifications for children and families, precipitating increased rates of FI. A recent study found that that the child tax credit, in addition to providing more economic stability for many low-income families, also reduced FI by 26% for families; if social policies can reduce FI (which, as suggested by our findings may be associated with increased prevalence and severity of chronic conditions), these same social policies may have the potential to reduce the burden of chronic conditions among children [30].

Our results also highlight the role of social and community risk factors (e.g., discrimination and neighborhood social support) on the prevalence and severity of selected chronic conditions. Experiencing discrimination was significantly associated with increased prevalence of each of the four chronic conditions but not with condition severity. Prior studies found strong evidence of racial disparities in ASD screening practices and in rates of connection to intervention services, and that Black and Hispanic children have lower rates of ASD but higher severity compared to White children [31]; our findings suggest that discrimination based on race and ethnicity may also play a role in determining severity of chronic conditions. Programs and intervention services for children with chronic conditions should be cognizant that racial and ethnic minority groups are more likely to experience discrimination, and such programs should incorporate trauma-informed and anti-racist care into their clinical practice [32]. Further research is needed to investigate how the concerted development and implementation of anti-racist approaches as best pediatric practice might enhance equity in pediatric health care, mitigate providers’ implicit biases, and—as our findings suggest—potentially reduce the prevalence and severity of asthma, ADHD, ASD, and overweight/obesity among children. Since 2020, the majority of US children are Black, Indigenous, or People of Color, making research that addresses race- and ethnicity-based discrimination a vital public health priority [33].

Prior studies have found that early environmental and epigenetic factors can lead to a variety of complex diseases and conditions in children, including the four conditions studied here [34, 35]. As such, addressing social risk factors in early childhood, prenatal care, and preconception care might prevent the development or reduce the severity of these childhood chronic conditions. Social risk factors are root causes of chronic disease, and disproportionately impact historically marginalized populations in the US. Investing in effective interdisciplinary SDOH interventions—especially those that promote economic stability and improve social, community, and neighborhood-level factors—may also reduce the prevalence and severity of asthma, ADHD, ASD, and overweight/obesity in childhood, though more translational research is needed to investigate this.

This study has several limitations. First, because we used cross-sectional data from the NSCH, we cannot infer causality, directionality, or temporality of the relationships. The results herein may be explained by the theory of social causation (i.e., poverty and social risks result in disease) and/or social selection (i.e., the challenges associated with managing a child’s chronic condition may lead to poverty and social risks) [36]; however, given the multitude of longitudinal studies and randomized controlled trials demonstrating temporal relationships between social risks and adverse health outcomes, we conceptualized our cross-sectional findings according to the social causation theory. Secondly, health information in the NSCH is based on caregiver recollection and is not independently verified, which may make these data susceptible to recall and selection bias. Additionally, children may be at genetic risk of one or more of the chronic conditions explored in this manuscript, though detailed data from NSCH respondents regarding genetic risks was not available in this dataset. Further, the cumulative impact of social risk factors on caregivers’ stress may have influenced their perception of severity of their child’s illness, exaggerating the relationships between social risks and severity of chronic conditions. Also, while we used insurance coverage as a measure for healthcare access, it is not a precise measure of actual healthcare use; the NSCH datasets analyzed herein changed items related to healthcare access and quality between 2017 and 2018, so these indicators could not be used in the analysis of the combined dataset. Future studies should consider exploring the influence of other measures of healthcare access and quality on the prevalence and severity of chronic conditions. Finally, the relationships presented herein are complex, and while our seven chosen risk factors represent the SDOH domains outlined in Healthy People 2030, there are many social risks not measured through NSCH and therefore not included in our analysis.

## Conclusions

This study elucidates relationships between several social risk factors and the prevalence and severity of common pediatric chronic conditions. Further research is needed to evaluate the causality of these relationships and to assess the role of protective factors and resiliency on the prevalence and severity of asthma, ADHD ASD, and overweight/obesity. Our findings suggest that mitigating social risks in childhood via social and health policies, pediatrics-based interventions, and integrated health-social care clinics may reduce the development and severity of chronic conditions among children.

## Data Availability

The datasets analyzed during the current study are available from the Data Resource Center for Child & Adolescent Health, https://www.childhealthdata.org/help/dataset.

## Abbreviations

ADHD: Attention deficit hyperactivity disorder
ASD: Autism spectrum disorder
BMI: Body mass index
FI: Food insecurity
NSCH: National Survey of Children’s Health
SDOH: Social determinants of health
US: United States
